# sBCMA Plasma Level Dynamics and Anti-BCMA CAR-T-Cell Treatment in Relapsed Multiple Myeloma

**DOI:** 10.3390/cimb44040098

**Published:** 2022-03-24

**Authors:** Katja Seipel, Naomi Porret, Gertrud Wiedemann, Barbara Jeker, Vera Ulrike Bacher, Thomas Pabst

**Affiliations:** 1 Department for Biomedical Research, University of Bern, 2008 Bern, Switzerland; 2 Department of Hematology and Central Hematology Laboratory, Inselspital, Bern University Hospital, University of Bern, 3010 Bern, Switzerland; NaomiAzur.Porret@insel.ch (N.P.); gertrud.wiedemann@insel.ch (G.W.); veraulrike.bacher@insel.ch (V.U.B.); 3 Department of Medical Oncology, Bern University Hospital, 3010 Bern, Switzerland; Barbara.Jeker@insel.ch

**Keywords:** B-cell maturation antigen (BCMA), soluble BCMA (sBCMA), multiple myeloma (MM), anti-BCMA CAR-T cell therapy

## Abstract

BACKGROUND: Novel chimeric antigen receptor T-cells (CAR-T) target the B-cell maturation antigen (BCMA) expressed on multiple myeloma cells. Assays monitoring CAR-T cell expansion and treatment response are being implemented in clinical routine. METHODS: Plasma levels of soluble BCMA (sBCMA) and anti-BCMA CAR-T cell copy numbers were monitored in the blood, following CAR-T cell infusion in patients with relapsed multiple myeloma. sBCMA peptide concentration was determined in the plasma, applying a human BCMA/TNFRS17 ELISA. ddPCR was performed using probes targeting the intracellular signaling domains 4-1BB und CD3zeta of the anti-BCMA CAR-T construct. RESULTS: We report responses in the first five patients who received anti-BCMA CAR- T cell therapy at our center. Four patients achieved a complete remission (CR) in the bone marrow one month after CAR-T infusion, with three patients achieving stringent CR, determined by flow cytometry techniques. Anti-BCMA CAR-T cells were detectable in the peripheral blood for up to 300 days, with copy numbers peaking 7 to 14 days post-infusion. sBCMA plasma levels started declining one to ten days post infusion, reaching minimal levels 30 to 60 days post infusion, before rebounding to normal levels. CONCLUSIONS: Our data confirm a favorable response to treatment in four of the first five patients receiving anti-BCMA CAR-T at our hospital. Anti-BCMA CAR-T cell expansion seems to peak in the peripheral blood in a similar pattern compared to the CAR-T cell products already approved for lymphoma treatment. sBCMA plasma level may be a valid biomarker in assessing response to BCMA-targeting therapies in myeloma patients.

## 1. Introduction

Multiple myeloma (MM) is a neoplasm of clonal plasma cells. MM is considered treatable, but incurable. Remission may be induced by treatment options involving steroids, chemotherapy, antibodies, targeted compounds, and autologous stem cell transplantation (ASCT). Advancements in treatments, including the introduction of immunomodulatory drugs (IMID), proteasome inhibitors (PI), and monoclonal antibodies have prolonged survival to a five-year survival rate of about 50% [1,2,3,4]. New therapies targeting BCMA are currently being investigated in clinical trials and will be incorporated into routine use, with the aim of further improving outcome rates.

The B-cell maturation antigen (BCMA, TNFRSF17) is a cell surface receptor of the TNF receptor superfamily preferentially expressed in malignant and normal plasma cells and mature B lymphocytes, specifically binding to TNFSF13B/TALL-1/BAFF (B-cell activating factor) and to various TRAF family members, and, thus, transducing signals for cell survival and proliferation [5,6]. Proteolytic shedding of the BCMA receptor reduces its cell-surface expression and the ligand-mediated survival of B cell subsets. This shedding is mediated by protease γ-secretase, and is partially dependent on ligand binding and receptor interactions. Shed receptors of soluble BCMA (sBCMA) may serve as biomarkers for auto-immunity and lymphoma [7]. sBCMA is shed from plasma cells, myeloma cells, and plasmacytoid dendritic cells. sBCMA peptide concentrations are elevated in MM plasma (median 500 ng/mL, range 100–1700 ng/mL) compared to healthy donors (median 40 ng/mL, range 10–80 ng/mL), with no difference between newly diagnosed patients and those with relapsed disease [8,9,10]. A remarkable decline in sBCMA levels was observed in patients with good responses to BCMA-targeted immunotherapy, suggesting sBCMA as a new biomarker for monitoring response to MM therapy [11]. In preclinical studies, anti-BCMA-CAR-T therapy showed low antigen-independent signaling and potent in vitro killing of myeloma tumor cells, across a range of BCMA expression levels, as well as sustained elimination of tumors and 100% survival, after single-dose administration in a mouse model of human multiple myeloma [12].

Idecaptagene-vicleucel (bb2121) is an anti-BCMA chimeric antigen receptor T-cell therapy for the treatment of multiple myeloma, approved by the FDA in 2021 for the treatment of adults with relapsed or refractory multiple myeloma, who have received at least three lines of anti-CD38/PI/IMID treatment. In clinical studies the median progression-free survival was 11.8 months in phase I [13] and 8.8 months in phase 2 trials [14]. In the absence of CAR-T therapy, RR/MM patients had a median PFS of 6.6 months and OS of 13.5 months [15]. Response to bb2121 is heterogeneous and often transient, possibly due to the presence of sBCMA in the plasma [7,9,11]. Serial sBCMA concentrations may decline more significantly in hematologic responders (PR/CR/sCR) than in non-responders (SD/PD) before day 28 post infusion [16].

The assays monitoring CAR-T expansion and assessing response to this novel therapeutic option await implementation into clinical routine. Here, we evaluate the first five patients with relapsed MM treated with anti-BCMA CAR-T cell therapy at the Insel-spital Bern, Switzerland.

## 2. Materials and Methods

In the work described here, we aimed to monitor response in anti-BCMA CAR T-cell recipients, by establishing a panel of laboratory assessments. We evaluated whether introducing routine laboratory parameters might facilitate timely identification of response and trigger therapeutic interventions. We established a digital droplet PCR (ddPCR) assay for CAR-T−specific T-cell receptor (TCR) measurement from peripheral blood (PB) and introduced sBCMA assessments at consecutive time points before and after CAR-T cell infusion.

### 2.1. Patients

The first five patients with relapsed/refractory multiple myeloma (RR/MM) receiving anti-BCMA CAR-T therapy at Bern University Hospital, Switzerland, were included in this study. They received their individual anti-BCMA CAR-T cell infusion between May and September 2021 after several lines of prior therapy. All patients gave written informed consent, and the study was approved by the local ethics committee of Bern, Switzerland (No. 2018-00628).

### 2.2. Lymphocyte Apheresis, Lymphocyte Depletion Chemotherapy, and CAR-T Infusion

Lymphocyte collections with the Spectra Optia (Terumo BCT) device were performed using the continuous mononuclear cell collection (CMNC) procedure. Peripheral CD3+ cell counts of blood and lymphocyte products were analyzed by multi-parameter flow cytometry (BD FACSCanto II). Five patients received idecabtagene vicleucel (ide-cel; previously bb2121). Ide-cel was manufactured following leukapheresis and then infused at dose levels aiming at 4.5E + 08 CAR+ T cells after 2 days interval, following lympho-depletion with 3 days of fludarabine 30 mg/m^2^ + cyclophosphamide 300 mg/m^2^.

### 2.3. Response Criteria

The criteria for assessing response were according to the International Myeloma Working Group [16,17]. Negative immuno-fixation on the serum and urine and disappearance of any soft tissue plasmacytomas and <5% plasma cells in bone marrow was required for CR response. In addition, normal free light chain (FLC) ratio and absence of clonal cells in the bone marrow by immunohistochemistry or immunofluorescence was required for stringent CR.

### 2.4. Establishment of ddPCR for CAR-T Quantification

In analogy to our previous study on CD19-CAR-T cell specific digital PCR [17], we designed a specific ddPCR assay to quantify sequences of the intracellular domain of the bb2121 CAR-T construct, using serial recipients’ peripheral blood samples. We designed primers and probes for the CAR-T transgenes to target the intracellular junction sequence between the effector (4-1BB) and co-stimulatory (CD3z) domains, similarly to Milone [18]. The procedure for the selection of the PCR primer sequences for quantitative assessment of the bb2121 CAR-T construct was performed following the strategy previously reported by Raje et al., with the primer sequences published there [12]. The reference gene was RPP30 (ribonuclease P protein subunit 30) [19].

### 2.5. Determination of sBCMA Plasma Levels in the Peripheral Blood

The Human BCMA/TNFRSF17 ELISA Kit (EH41RB, Thermo Fisher Scientific, Waltham, MA, USA) is a solid-phase sandwich enzyme-linked immunosorbent assay (ELISA) designed to detect and quantify the level of human BCMA in cell culture supernatants, plasma, and serum. ELISA assay was done according to the manufacturer’s instructions using BCMA protein standard. sBCMA levels were represented as the mean of triplicate samples for each specimen.

## 3. Results

**Figure 1:**
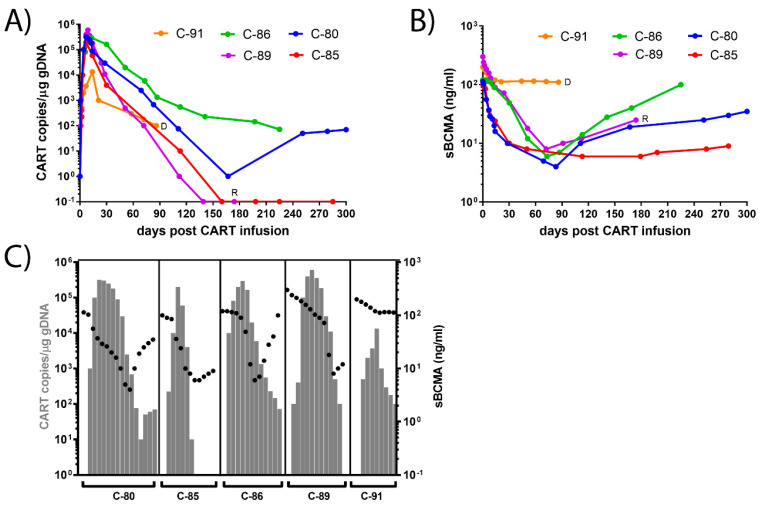
Dynamics of CAR-T and sBCMA plasma levels in the peripheral blood of patients. (**A**) Dynamics of CAR-T copies/μg genomic DNA in the peripheral blood. Rapid rise to high peak levels (>1.9E + 05 copies/μg), followed by decline to undetectable levels (C-85, C-89) or persistence at low levels (C-80, C-86). Rapid rise to mediocre peak levels (1.3E + 04 copies/μg) and decline to low levels (C-91). The concentration of the CAR-T copies/μg gDNA is given on a logarithmic scale on the y-axis. (**B**) Dynamics of sBCMA concentrations in the plasma. Rapid decline to minimal levels (C-80, C-85, C-86, C-89), followed by rebound to normal levels (C-80, C-86). Persistence of elevated levels (C-91). Response determination at the end of the study entailed three sCR (C-80, C-85, C-86), one relapsed (C-89, R), one deceased (C-91, D). The concentration of the sBCMA is given on a logarithmic scale on the y-axis. (**C**) Dynamics of CART copies/μg gDNA (grey bars) and sBCMA (ng/mL) in the plasma (black dots).

We evaluated the first five myeloma patients treated with bb2121 CAR-T cell therapy at a single academic center (Table 1). After CAR-T cell administration, patients can develop specific acute toxicities, including cytokine release syndrome (CRS) or immune effector cell-associated neurotoxicity syndrome (ICANS) [20], with up to 40% of patients requiring ICU admission. Three of the five patients in our cohort developed CRS grade 1 or 2, and two patients had ICANS grade 1 or 2. Response determination entailed measurement of standard myeloma blood parameters every three weeks. Four patients achieved a complete remission (CR) in the bone marrow one month after CAR-T infusion, with three patients achieving stringent CR, as determined by flow cytometry techniques. bb2121 CAR-T DNA was detectable in peripheral blood for up to 300 days, with copy numbers peaking 7 to 14 days post infusion (Figure 1A). sBCMA plasma levels started dropping 1–10 days post infusion and reached minimal levels 60 to 80 days post infusion (Figure 1B). In three patients, sBCMA levels started to rebound immediately after the CAR-T levels declined. In one patient, high levels of sBCMA were consistently detected after the initial reduction, and this patient died of progression 100 days post CAR-T cell infusion.

### 3.1. Patients Characteristics

Five patients with relapsed/refractory multiple myeloma were included in this study (Table 1). They received anti-BCMA CAR-T cells after two to five lines of prior therapy. Response determination entailed detection of standard myeloma blood parameters every three weeks, according to the international myeloma working group [21]. The CAR-T treatment resulted in complete remission (CR) in four patients with CAR-T peak levels exceeding 2E + 05 copies/μg gDNA and sBCMA plasma concentration dropping to minimal levels (<10 ng/mL) within 90 days post infusion.

Patient 1 (C-80), first diagnosed with multiple myeloma, stage III (R-ISS) received three lines of prior therapy and autologous stem cell transplant (ASCT). After the third relapse, anti-BCMA CAR-T treatment resulted in stringent complete remission four weeks after CAR-T cell infusion. CAR-T copy number in the peripheral blood peaked on day 7 at high levels (>3E + 05 copies/μg gDNA), followed by decline to minimal levels after six months, then remained at low levels (100 copies/μg gDNA) until end of observation. sBCMA plasma levels, initially at 115 ng/mL, started dropping immediately after infusion, and reached minimal levels (5 ng/mL) eleven weeks after infusion. sBCMA plasma levels started to rise to normal levels 4 months after infusion, and 10 months after infusion the response status was still sCR.

Patient 2 (C-85), first diagnosed with multiple myeloma, stage II (R-ISS), had received two lines of prior therapy and ASCT. After second relapse, anti-BCMA CAR-T treatment resulted in stringent complete remission four weeks after CAR-T cell infusion. CAR-T copy number in the peripheral blood peaked on day 7 at intermediate levels (2E + 05 copies/μg gDNA) and subsided below the detection limit after five months. sBCMA plasma levels, initially at 100 ng/mL, started dropping immediately after CAR-T cell infusion, and reached minimal levels (10 ng/mL) four weeks after infusion, persisting below normal levels for nine months when the response status was still sCR.

Patient 3 (C-86), first diagnosed with multiple myeloma, stage II (R-ISS) received five lines of prior therapy and ASCT. After second relapse, anti-BCMA CAR-T treatment resulted in complete remission four weeks after infusion. CAR-T copy number in the peripheral blood peaked on day 13 at high levels (3.2E + 05 copies/μg gDNA) and was still at 128 copies/μg gDNA after six months. sBCMA plasma levels, initially at 120 ng/mL, started dropping 10 days after infusion, and reached minimal levels (10 ng/mL) two months after CAR-T cell infusion. sBCMA plasma levels started to rise above normal levels (rebound) three months after infusion. Eight months after CAR-T infusion sBCMA plasma concentration had reached pretreatment levels, while the response status was still CR.

Patient 4 (C-89) had received two lines of prior therapy, but was not eligible for ASCT. After second relapse anti-BCMA CAR-T, therapy resulted in complete remission four weeks after infusion. CAR-T copy number in the peripheral blood peaked on day 9 at high levels (6E + 05 copies/μg gDNA) and subsided below the detection limit after five months. sBCMA plasma levels, initially at 280 ng/mL, started dropping immediately after infusion and reached minimal levels two months after CAR-T cell infusion. Six months after CAR-T infusion, the response determination indicated a relapse.

Patient 5 (C-91), first diagnosed with multiple myeloma, stage II (R-ISS), had received two lines of prior therapy and ASCT. Anti-BCMA CAR-T treatment resulted in stable disease (SD) four weeks after infusion. CAR-T copy number in the peripheral blood peaked on day 14 at low levels (1.3E + 04 copies/μg gDNA) and was at 100 copies/μg gDNA after three months when disease was progressive (PD). sBCMA plasma levels, initially at 200 ng/mL, started dropping immediately after infusion; however, they soon reached a plateau at 120 ng/mL. The disease progressed, and the patient died 100 days after CAR-T infusion.

### 3.2. Dynamics of CAR-T Concentration in the Peripheral Blood

We determined the dynamics of CAR-T cell presence in the peripheral blood by evaluating the copy number, as well as persistence after infusion, similarly to the analysis done in the phase I and phase II studies [13,14]. Expansion of CAR-T cells peaked above 2–E + 05 copies/μg gDNA in the four patients with good responses, and at 1.3E + 04 copies/μg DNA) in the one patient with partial response (Figure 1A). Three patterns of dynamics were observed: 1. Rapid increase to high peak levels (>1.9E + 05 copies/μg), followed by decline to undetectable levels (C-85, C-89). 2. Rapid increase to high peak levels, followed by decline with persistence at low levels (C-80, C-86). 3. Rapid increase to mediocre peak levels (1.3E + 04 copies/μg), followed by decline with persistence at low levels (C-91). CAR-T cells were present for less than six months after infusion in two patients (C-85, C-89), and over six months in two patients (C-80, C-86).

### 3.3. Dynamics of sBCMA Plasma Levels

**Figure 2:**
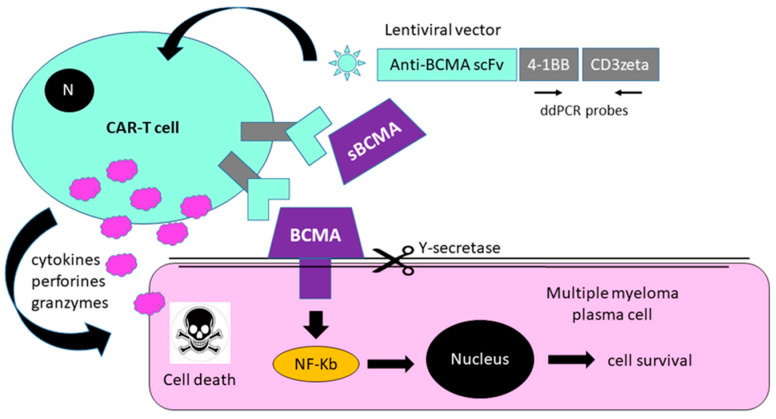
Anti-BCMA CAR-T cell treatment. CAR-T cells target the BCMA protein present on the surface of the myeloma cells and release cytokines, perforines, and granzymes. BCMA shedding is partially dependent on ligand binding and receptor interactions. sBCMA may act as a decoy blocking the CAR-TCR; thus, leading to reduced efficacy of anti-BCMA-CAR-T therapy.

Proteolytic shedding of the BCMA receptor reduces its cell-surface expression and ligand-mediated survival of B cell subsets. This shedding is mediated by protease γ-secretase, and is partially dependent on ligand binding and receptor interactions. sBCMA plasma levels are elevated in MM sera (100–300 ng/mL) compared to healthy donors (10–80 ng/mL), with no difference between newly diagnosed patients and those with relapsed disease [8,9,10]. A remarkable decrease in sBCMA level was previously observed in patients with good responses to BCMA-targeted immunotherapy, suggesting sBCMA as a suitable biomarker to assess response to MM therapy [11]. Here, we assessed soluble BCMA as a serum-based universal marker of myeloma burden, and correlated sBCMA levels with response duration. sBCMA plasma levels started dropping 1–10 days post infusion, and minimal levels (<10 ng/mL) were reached 60 to 80 days post-infusion (Figure 1B,C). After the initial decline, sBCMA levels started to rise again (rebound), reaching normal levels in three cases, and rising to pretreatment levels in one patient eight months after CAR-T cell infusion. Four patterns of dynamics of sBCMA levels were observed: 1. Rapid decline to minimal levels, followed by rebound to normal levels (C-80, C-89). 2. Rapid decline to minimal levels with persistence (C-85). 3. Rapid decline to minimal levels, followed by rebound to normal levels and rise to pretreatment levels (C-86). 4. Persistently high sBCMA plasma levels (C-91). Shedding of BCMA may reduce the efficacy of ide-cel CAR-T treatment, since the target is no longer present on the target cell, and sBCMA can act as a decoy that neutralizes the compound (Figure 2).

## 4. Discussion

Four of the first five patients receiving anti-BCMA CAR-T cells at the University Hospital, Bern, demonstrated favorable response to anti-BCMA CAR-T treatment. Complete remission (CR) in the bone marrow was prevalent in four patients (80%) one month post CAR-T infusion, with stringent CR (sCR) present in three patients (60%), as determined by flow cytometry techniques. This is in accordance with results from a phase II study [14], where, at the target dose of 4.50E + 08 CAR-T cells, a response was observed in 44 of 54 patients (81%), and a complete response or better was observed in 21 of 54 patients (39%). Anti-BCMA CAR-T cell expansion appeared to peak in the peripheral blood in multiple myeloma patients, with a similar pattern compared to CAR-T cell products already approved for commercial use in lymphomas [22,23].

Responses to anti-BCMA CAR-T treatment in this small cohort were found to associate with peak expansion by qPCR (>2E + 05 copies/μg DNA for CR vs. 1E + 04 copies/μg for PR/SD), similarly to data reported by Cohen et al., 2019 [16], who described a significant association of response with peak expansion by qPCR (median 7E + 05 copies/μg DNA for ≥PR vs. 6E + 03 copies/μg for <PR), as well as with persistence over the first 28 days. CAR-T cells were detectable for less than six months in two patients, and for up to 10 months in two patients, at low levels. A similar duration was described in the cellular kinetic analysis of the phase II trial where CAR-T cells were present in 59% of patients at 6 months and in 36% at 12 months after infusion [14]. The low level presence of CAR-T cells (100 copies/μg gDNA) may be insufficient to guard against myeloma recurrence. In addition, several months after infusion, the CAR-T cells may become functionally compromised and the myeloma cells may become resistant to the CAR-T cells.

With respect to sBCMA levels, a clinical reference value has not been defined. However, sBCMA peptide concentrations are elevated in MM plasma (100–1000 ng/mL) compared to healthy donors (10–80 ng/mL) [14,22,23]. In four of the five MM patients in our study, sBCMA concentrations in the plasma, at 100–300 ng/mL before CAR-T infusion, declined to minimal levels below 10 ng/mL within 90 days post anti-BCMA CAR-T infusion, before rebounding to normal levels. Higher baseline sBCMA of 300 ng/mL was detected in two patients, one without clinical response and one with good initial clinical response but early relapse, indicating that higher baseline sBCMA may affect therapeutic efficacy, possibly by blocking the chimeric T cell receptors of the effector cells.

sBCMA levels above normal levels after the decline of CAR-T cells may indicate the impending end of remission. sBCMA plasma concentrations of 100 ng/mL can represent normal levels in one patient, and indicate recurrence of myeloma in another patient. sBCMA plasma levels may be easily determined together with standard remission controls on a monthly basis and can serve, not only as response monitoring tool, but also as an early predictor of response.

A remarkable decrease in sBCMA level was observed in the four patients with good responses to bb2121 therapy. With this limited number of patients, definite conclusions on the utility of sBCMA as a response monitoring marker are not possible. However, the study may present a proof of principle, suggesting that sBCMA may represent a promising biomarker of response to BCMA-targeted immunotherapy. sBCMA concentrations were found to decline more significantly after CAR-T cell infusions in responders (CR/sCR) than in non-responders (SD). sBCMA was detectable in all five patients at the end of the study, suggesting that BCMA antigen loss is not a prevalent mechanism of escape from ide-cel therapy. sBCMA concentrations remained at low levels in long-term responders, confirming sBCMA plasma levels as a useful adjunctive biomarker for assessing myeloma disease response and progression. Larger studies are required to evaluate the prognostic significance of sBCMA plasma levels for their potential as biomarker of response and predictor of response to BCMA-targeting therapies in relapsed multiple myeloma patients.

Identification of the observed CAR-T copy number kinetics suggests investigation of whether the pattern ‘rapid rise to high peak levels, followed by decline, with persistence at low levels’ may be associated with superior efficacy of CAR-T treatment. Identification of the observed sBCMA plasma level dynamics suggests further investigation of whether the pattern ‘rapid decline to minimal levels < 10 ng/mL’, as well as ‘duration at minimal levels’, may be associated with superior efficacy of CAR-T treatment. These questions can be addressed once the therapy is approved and more MM patients can be admitted to BCMA CAR-T therapy.

## Figures and Tables

**Table 1:** R/R MM Patient characteristics.

ID	C-80	C-85	C-86	C-89	C-91	Average (Range)
age at diagnosis (years)	63	58	55	69	45	58 (45–69)
stage R-ISS	III	II	II	na	II	
plasma cell infiltration (%)	85	50	80	90	60	73 (50–90)
FISH	t(11;14)	t(4;14)	t(11;14)/+1q	na	t(4;14)/+1q	
IgA (g/L)		22	50	46		
light chain kappa (mg/L)		23		6	223	
light chain lambda (mg/L)	13,400		382	604	11	
lines of prior therapy	3	2	5	2	3	3 (2–5)
prior ASCT	1	1	1	0	1	0.8 (0–1)
number of relapses	3	2	2	2	0	2 (0–3)
time to CART (years)	7	6	6	1	1	4.2 (1–7)
CAR-T peak (copies/μg gDNA)	3.2E + 05	2.0E + 05	3.0E + 05	6.0E + 05	1.3E + 04	2.9E + 05
CAR-T peak day post infusion	7	7	13	9	14	10 (7–13)
sBCMA pre-infusion (ng/mL)	115	100	120	280	200	163 (100–280)
sBCMA 4 weeks post infusion	10	10	50	60	120	50 (10–120)
sBCMA 8 weeks post infusion	5	5	12	15	115	33 (5–115)
status, 4 weeks post infusion	sCR	sCR	sCR	CR	SD	
status, 8 weeks post infusion	sCR	sCR	sCR	CR	PD	
status, 6 months post infusion	sCR	sCR	sCR	relapse	deceased	

Abbreviations: relapsed/refractory (R/R); multiple myeloma (MM); autologous stem cell transplant (ASCT); complete remission (CR); stable disease (SD); progressive disease (PD).

## Data Availability

Data is contained within the article.
